# Artificial intelligence-based automatic assessment of lower limb torsion on MRI

**DOI:** 10.1038/s41598-021-02708-y

**Published:** 2021-12-01

**Authors:** Justus Schock, Daniel Truhn, Darius Nürnberger, Stefan Conrad, Marc Sebastian Huppertz, Sebastian Keil, Christiane Kuhl, Dorit Merhof, Sven Nebelung

**Affiliations:** 1 Department of Diagnostic and Interventional Radiology, University Hospital Düsseldorf, Düsseldorf, Germany; 2 Institute of Imaging and Computer Vision, RWTH University Aachen, Aachen, Germany; 3 Department of Diagnostic and Interventional Radiology, Aachen University Hospital, Pauwels Street 30, 52074 Aachen, Germany; 4 Institute of Informatics, Heinrich Heine University, Düsseldorf, Germany

**Keywords:** Musculoskeletal system, Bone, Computational science, Software

## Abstract

Abnormal torsion of the lower limbs may adversely affect joint health. This study developed and validated a deep learning-based method for automatic measurement of femoral and tibial torsion on MRI. Axial T2-weighted sequences acquired of the hips, knees, and ankles of 93 patients (mean age, 13 ± 5 years; 52 males) were included and allocated to training (*n* = 60), validation (*n* = 9), and test sets (*n* = 24). A U-net convolutional neural network was trained to segment both femur and tibia, identify osseous anatomic landmarks, define pertinent reference lines, and quantify femoral and tibial torsion. Manual measurements by two radiologists provided the reference standard. Inter-reader comparisons were performed using repeated-measures ANOVA, Pearson’s *r,* and the intraclass correlation coefficient (ICC). Mean Sørensen-Dice coefficients for segmentation accuracy ranged between 0.89 and 0.93 and erroneous segmentations were scarce. Ranges of torsion as measured by both readers and the algorithm on the same axial image were 15.8°–18.0° (femur) and 33.9°–35.2° (tibia). Correlation coefficients (ranges, .968 ≤ r ≤ .984 [femur]; .867 ≤ r ≤ .904 [tibia]) and ICCs (ranges, .963 ≤ ICC ≤ .974 [femur]; .867 ≤ ICC ≤ .894 [tibia]) indicated excellent inter-reader agreement. Algorithm-based analysis was faster than manual analysis (7 vs 207 vs 230 s, *p* < .001). In conclusion, fully automatic measurement of torsional alignment is accurate, reliable, and sufficiently fast for clinical workflows.

## Introduction

Torsional deformities of the lower limbs are defined as abnormal rotation of the proximal bone axis versus the distal bone axis and may adversely affect joint health directly or indirectly. In addition to hip, knee, and ankle pain, torsional deformities predispose to gait abnormalities such as “in-toeing” or “out-toeing” and are associated with tripping, patellar malalignment and dislocation as well as other osseous deformities that may persist into adulthood^1^. Moreover, torsional deformities have been implicated in the etiopathogenesis of slipped capital femoral epiphysis^2^, femoroacetabular impingement^3^, Legg–Calvé–Perthes disease^4^, developmental dysplasia of the hip^5^, and osteoarthritis^6^.

Exact quantification of torsional deformities is a prerequisite for successful operative treatment and favourable long-term outcomes^7^. Originating in the 1970s, torsional alignment has traditionally been measured by computed tomography (CT)^8^. To reduce radiation exposure to the paediatric patient population, Magnetic Resonance Imaging (MRI) techniques have evolved^9^ to be diagnostically equivalent with traditional CT techniques while fitting into tight clinical schedules^10,11^.

For both CT and MRI, accuracy and reliability are challenged by substantial intra- and inter-reader variability that may be as high as 10.8° and 15.6°, respectively^12^, which may be largely attributed to inconsistent level, obliquity, and method of selecting the respective reference lines^8,10^. Yet, even with *à-priori* selected axial or oblique images, the variability persisted^8,13^, highlighting the difficulty of correctly and reliably identifying pertinent axes in 3D objects (such as bone) using 2D images.

In this era of much-sought standardization, there is a clear need for standardized, accurate, and reproducible evaluation of lower limb torsion that may be addressed by deep learning. Deep learning techniques refer to a subtype of machine learning that rely on computational networks to learn from image data by progressively extracting higher-level features without manual engagement. Such techniques have been applied across the disciplines, including medical image processing^14^. In musculoskeletal radiology, the diagnostic possibilities of deep learning are ample and include image reconstruction, image data transformation, tissue segmentation, workflow support, opportunistic screening, compositional analysis, and disease detection^15–21^. To our knowledge, deep learning techniques have not been studied as a diagnostic support system for the assessment of torsional alignment. Against this background, this study’s purpose was to develop, train, and validate a deep learning-based diagnostic support system for automatic segmentation and post processing of torsional alignment on MRI. Our hypotheses were that (i) lower limb torsion may be accurately and automatically determined on clinical MRI studies of the hips, knees, and ankles and that (ii) the torsional angles thus obtained are as accurate as those determined manually by radiologists yet at a fraction of the time demand.

## Results

### Patient characteristics

Complete MRI studies of 93 patients were included. Patients were children, teenagers, and young adults (mean age, 13.1 years ± 5.0 [standard deviation]; range 5–34 years; 52 males). After their random allocation to the training (*n* = 60), validation (*n* = 9), and test sets (*n* = 24), demographic details of the sets were largely similar (Supplementary Table 1).

### Segmentation performance

**Figure 1:**
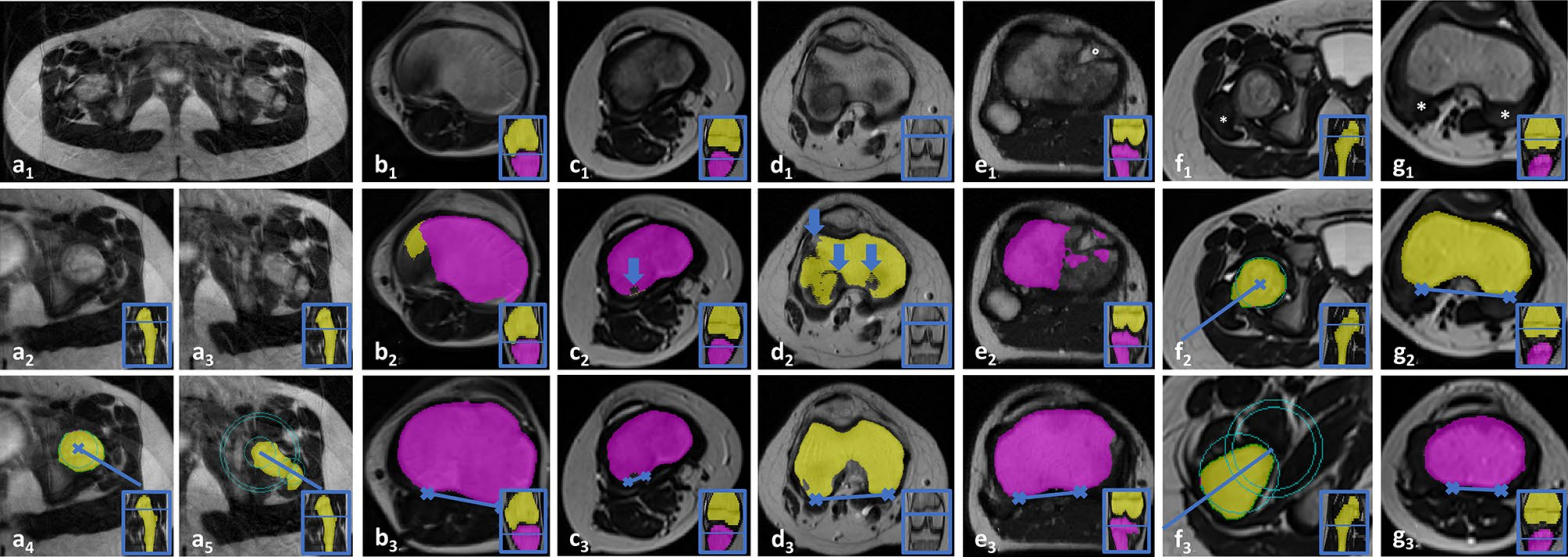
Representative cases that challenged correct segmentation and determination of reference lines. (**a**) Motion artefacts during the pelvic MRI scan (a_1_) caused smearing of the images at the level of the femoral head (a_2_) and neck (a_3_), erroneous delineation of the femoral head contour (a_4_), and incorrect identification of the femoral neck axis through the greater trochanter (a_5_). (**b**) Motion artefacts during the MRI scan of the knee (b_1_) created an artificial protrusion of the medial femur (b_2_) that did not negatively affect the reference line (b_3_). (**c**, **d**) Prominent and variable epiphyseal plates at the tibia (**c**) or femur (**d**) were associated with inaccurate segmentations (block arrows in c_2_, d_2_) that caused misplacement of the reference line (c_3_) or not (d_3_). (**e**) Post-surgical changes and fat graft interposition at the anteromedial tibia (°) after physiolysis (e_1_) caused inaccurate segmentation (e_2_) but did not negatively affect the reference line (e_3_). (**f**) At the hip, immature bone characterized by the cartilaginous greater trochanter and femoral neck (* in f_1_) is segmented incorrectly (f_2_) and the femoral neck axis is identified too caudal (f_3_). (**g**) Similarly, cartilaginous femoral and tibial condyles (* in g_1_) are characzterized by incorrect segmentations and reference lines (g_2_, g_3_). Inset coronal images framed in blue indicate the height of the axial slices. Patient age and gender: 9 years and male (**a**, **c**), 11 years and male (**b**, **e**), 12 years and male (**d**), 5 years and male (**g**).

Similarity of the automatic and manual segmentation outlines was evaluated on the patients of the test set with two lower limbs each. Mean Sørensen-Dice coefficients to quantify similarity between manually and automatically segmented femoral and tibial outlines were 0.92 ± 0.02, 0.93 ± 0.03, 0.93 ± 0.02, 0.93 ± 0.02, and 0.89 ± 0.02 for the proximal femur, the distal femur, the proximal tibia, the distal tibia, and the distal fibula, respectively, indicating excellent correspondence in all anatomic regions. Yet, erroneous segmentations occurred if (i) image quality was poor secondary to motion artefacts (Fig. 1a_1-5_, b_1-3_), if (ii) epiphyseal plates were prominent and heterogeneous (Fig. 1c_1-3_, d_1-3_), or if (iii) post-surgical changes were present, e.g., after physiolysis (Fig. 1e_1-3_). Erroneous segmentation outlines were also found in the presence of immature (pre-formed) bone (Fig. 1f_1-3_, g_1-3_). In some instances, incorrect segmentations affected the definition of the reference lines (Fig. 1a_1-5_, c_1-3_, f_1-3_, g_1-3_). At the ankle, all segmentations (48/48 joints) were correct, while at the knee (41/48 joints) and hip (42/48 joints), segmentations were partially incorrect because of excessive motion artefacts (3 joints), variable bone maturation (8 joints), post-surgical status (1 joint), and inhomogeneous bone texture (1 joint).

### Quantification of femoral and tibial torsion

Mean values of femoral and tibial torsion are indicated in Table 1 and Supplementary Table 2. Mean femoral torsion as determined by both radiologists varied between 16.1° (Lee method, R1) and 32.2° (Murphy method, R1), while the algorithm quantified mean femoral torsion as 15.8°. Mean tibial torsion was equally variable and ranged from 20.9° (talus method, R1) to 34.3° (ellipses method, R2), with 35.2° determined by the algorithm. Post-hoc testing revealed significant differences between the algorithm and both radiologists for all femoral and tibial methods except for the Lee (Alg vs. R1 [*p* = 0.859]) and ellipses methods (Alg vs R1 [*p* = 0.212]; Alg vs R2 [*p* = 0.622]).

**Table 1:** Mean femoral and tibial torsion as determined by the Lee and ellipses methods.

	Method	Reader			*p*-values	Post-hoc test details		
		R1	R2	Alg		Alg vs R1	Alg vs R2	R1 vs R2
Femoral torsion	Lee	16.1 (11.6; 20.6)	18.0 (13.2; 22.9)	15.8 (11.0; 20.6)	< 0.001	0.859	** < 0.001**	** < 0.001**
Tibial torsion	Ellipses	33.9 (30.8; 37.0)	34.3 (30.7; 37.9)	35.2 (31.7; 38.6)	0.311	0.212	0.622	0.877

Readers are radiologist 1 (R1), radiologist 2 (R2), and the algorithm (Alg). Means (95% confidence intervals) [°]. Statistical analysis was performed by repeated measures ANOVA followed by pair-wise Tukey's post-hoc test. Results of the other methods of determining femoral (according to the Reikeras, Tomczak, and Murphy methods) and tibial torsion (according to the bimalleolar and talus methods) are detailed in Supplementary Table 2. Adjusted *p*-values with significant differences indicated in bold type.

### Inter-reader agreement and differences

The absolute inter-reader differences between R1 or R2 and the algorithm are detailed in Table 2 and Supplementary Table 3. Because the algorithm was implemented in close resemblance with the Lee and ellipses methods, the absolute inter-reader differences were lowest and the inter-reader correlations (0.968 ≤ *r* ≤ 0.971) and ICCs (0.963 ≤ ICC ≤ 0.966) were highest for these two methods. With increasing distance to the axial image used for the algorithmic definition of the reference lines, absolute differences increased, and inter-reader ICCs decreased with lowest ICCs found for the Tomczak, Murphy, bimalleolar, and talus methods. Irrespective of the method used for defining the reference lines, inter-reader correlations between R1 or R2 and the algorithm were very strong ^22^ as indicated by 0.854 ≤ *r* ≤ 0.971. Between both radiologists, inter-reader agreement was excellent for femoral torsion (ICCs ≥ 0.976) and very high for tibial torsion (0.871 ≤ ICC ≤ 0.933), with corresponding mean absolute differences ranging from 2.6° to 3.2° and from 3.3° to 5.0°.

**Table 2:** Metrics of inter-reader difference and agreement for femoral and tibial torsion as determined by the Lee and ellipses methods.

	Method	Comparison	Absolute difference [°]		Pearson's r		ICC	
			R2	Alg	R2	Alg	R2	Alg
Femoral torsion	Lee	R1	2.6 (1.9; 3.3)	3.3 (2.5; 4.0)	0.984 (< 0.001)	0.968 (< 0.001)	0.974 (0.96, 0.99)	0.963 (0.93; 0.98)
		R2	na	3.7 (3.0; 4.5)	na	0.971 (< 0.001)	na	0.966 (0.94; 0.98)
Tibial torsion	Ellipses	R1	5.0 (4.0; 6.0)	4.2 (3.2; 5.1)	0.871 (< 0.001)	0.904 (< 0.001)	0.865 (0.77; 0.92)	0.867 (0.78; 0.92)
		R2	na	5.1 (4.1; 6.2)	na	0.867 (< 0.001)	na	0.894 (0.82, 0.94)

Readers were the two radiologists (radiologist 1 [R1], radiologist 2 [R2]) and the algorithm (Alg). In a pair-wise manner, absolute differences [°] and agreement were quantified in terms of Pearson’s correlation coefficient *r* and the intraclass-correlation-coefficient (ICC, single scorings [not adjusted]). Means (95% confidence intervals). na – not applicable. Results of the other methods of determining femoral (according to the Reikeras, Tomczak, and Murphy methods) and tibial torsion (according to the bimalleolar and talus methods) are detailed in Supplementary Table 3.

Supplementary Figure 1 visualizes inter-reader differences as a function of their means. No systematic bias in data distribution was observed.

### Time demand

On average, the algorithm-based analysis of femoral and tibial torsion of one lower limb took 7 s on the specialized workstation, which was significantly faster than the two radiologists (R1: 207 ± 17 s; R2: 230 ± 18 s; *p* < 0.001).

## Discussion

In this study, we implemented an algorithm that automatically analyses lower limb torsion based on MRI. The algorithm performs well in segmenting bone outlines, in identifying relevant anatomy, and in quantifying torsion and thereby renders the automatic measurements of torsional alignment accurate, reliable, stable, and fast for clinical workflows.

Recently, applications of machine learning have exploded across the disciplines, including musculoskeletal radiology, where they are intended to improve clinical workflows^23^. Analysis of torsion seems well suited because image acquisition is standardized^9^, clinical relevance is pronounced^3^, and manual reference methods are variable^8,10^.

With regards to clinical experience and requirements, we intentionally implemented an analysis pipeline of sequential segmentation and post-processing to allow the radiologist to review (and confirm) the outputs. For **segmentations**, we used a modified, yet conventional U-net convolutional neural network and found excellent segmentation accuracy as indicated by mean Sørensen-Dice coefficients of 0.89–0.93, which is comparable with *state-of-the-art* techniques^24^. Methodologically, the focus on the Sørensen-Dice coefficient may be justified, yet merely numeric considerations fall short on reflecting the algorithm’s performance in challenging anatomies. Segmentations were largely correct yet challenged by poor image quality (due to motion artefacts), heterogeneous epiphyseal plates (due to partial volume effects), post-surgical changes, and immature bone. These conditions were prone to inaccurate segmentations (and, subsequently, inaccurate reference lines) and may be improved by (i) increasing the number and diversity of image datasets, (ii) implementing pre-defined bone-shape models, and (iii) strictly controlling image quality. Including more MRI studies with variable (and dysplastic) limb shapes into the training set will most likely improve the segmentation performance. Yet, not least due to ordering practices of referring orthopedists, overall numbers of torsional MRI studies for adolescents remain low, not just at our institution^10,11^. Including active shape or appearance models may help in generating plausible segmentations. Traditionally, these models have used pre-defined bone shapes that undergo iterative deformation to fit the actual to the ideal bone contours, yet necessitate substantial manual pre-processing^25^. Recent approaches combine shape models with deep-learning techniques^26,27^ and may provide viable amendments to our method. Including control mechanisms of image quality may help exclude MRI studies with poor image quality, yet, for the time being, remain the radiologist’s responsibility.

Subsequent **algorithmic post-processing** was adapted to the specific anatomy. At the hip, the peripheral femoral neck was automatically identified and used as the proximal reference line. Our method is closest to the method of Lee^28^ who also used the most proximal axial image (where the femoral neck is narrowest) to connect the centres of the femoral head and neck. While our method identified the femoral head to locate the peripheral femoral neck, Lee used it as part of the reference line. Also, our method defined the femoral neck axis as the reference line, whereas Lee estimated the femoral neck centre as part of the reference line. Despite these differences, both methods were numerically closely related in terms of mean torsion angles, absolute differences, and inter-reader correlations. Notably, inter-method variability was comparable to inter-reader variability, which underpins the method’s reliability. Alternative manual reference methods according to Reikeras, Tomczak, or Murphy^29–31^ use more distal axial images for the proximal reference line. Because the distal femoral neck is more posterior (versus the proximal femoral neck), femoral torsion was higher and significantly different from our method. Nonetheless, inter-method correlation in terms of Pearson’s *r* was excellent, irrespective of the reference method, while ICCs gradually decreased. In our study, Pearson’s *r* seems the more intuitive measure of association than the ICC, because each value is scaled by its own mean and standard deviation for Pearson’s *r*, while all data are pooled for the ICC^32^.

Around the knee, the most posterior extensions of the medial and lateral condyles were identified on the axial images with the largest convex bone areas and connected as the reference lines. While referencing to the posterior condyles is largely consented^8–11,28,33^ and highly reproducible^8,29^, some discrepancies remain on the exact definition of the axial plane^34^. Our method lends itself more to automation than others that involve distance measurements and anatomic considerations^11,34^.

At the ankle, the tibial and fibular centroids were connected as the reference line. Automatically defining the centroids proved reliable and did not result in erroneous segmentations. However, this method may lose accuracy in cases with abnormal or displaced fibulae^35^. Numerically, our method was related closest to the ellipses method that connects the centers of two ellipses placed along the medial malleolus and the fibular notch^8,36^. This finding is plausible as both methods use similar axial images, yet inter-reader agreement was slightly lower than for femoral torsion. Likely, fitting ellipses to non-spherical geometries to define their centers introduces more variability than determining the centroids directly. Alternative manual reference methods such as the bimalleolar and talus methods used more distal axial images^8,37^ and were characterized by lower tibial torsion. In line with earlier studies^8,34,35^, these findings reflect the different axes of the talocrural joint^38^ and highlight the value of methodologic consistency. Most likely, the lower ICCs (and very high Pearson’s *r*) for the distal methods are indicative of differences in reference images, lines, and anatomy.

This study has **limitations**. First, patient numbers were limited which is mainly due to conservative ordering practices that is still heavily reliant on CT and reduces the availability of MRI studies. Second, we only assessed T2-weighted sequences that had been acquired using a specific scanner-coil-configuration at a single site, while other institutions may use different settings so that variability across vendors, platforms, and sequences remains unaccounted for. Consequently, the algorithm’s generalizability (and multi-institutional validation) remain to be addressed. Third, even though consecutive patients from the clinical routine were included (and no patients were excluded for medical reasons), our results relate to younger patients with non-traumatic torsional deformities only, while post-traumatic conditions were not assessed. Fourth, alternative deep learning-based methods that derive predictions of torsional alignment directly from the MR images (without necessitating additional bone segmentation, landmark identification, and reference line definition as intermediate steps) were not evaluated and may improve predictions.

In **conclusion**, femoral and tibial torsion can be automatically assessed on clinical MR images of the hip, knee, and ankle joints based on U-net convolutional neural networks for segmentation and algorithm-based analyses of anatomic landmarks and reference lines. The developed methodology is sufficiently accurate, reliable, and fast to enhance clinical workflows.

## Materials and methods

### Study design

This is a retrospective single-center comparative imaging study that was conducted in accordance with local data protection regulations. The local ethical committee (Ethical Committee of the Medical Faculty, RWTH [Rheinisch-Westfälische Technische Hochschule] Aachen University, reference no. 028/19) approved the study and waived the requirement to obtain individual informed consent. All pediatric and adult patients who had undergone torsional MRI, consisting of stacks through the pelvis, both knees, and both ankles, between July 2013 and August 2020 were identified by a database query. Of the 107 patients thus identified, 14 were excluded because at least one joint was missing (*n* = 8) or motion artefacts were excessive (*n* = 6). The flowchart is visualized in Supplementary Figure 2. Otherwise, no further exclusion criteria were defined and patients with skeletal dysplasia, other skeletal abnormalities, and orthopedic hardware were included to reflect clinical reality. Eventually, datasets of 93 patients were included and randomly allocated to strictly separated training (*n* = 60, 65%), validation (*n* = 9, 10%), and test sets (*n* = 24, 26%). On the training set, the algorithms were developed and trained to perform image segmentation and post processing, while on the validation set, the optimal hyperparameters that determine the network’s structure and training were validated. On the test set, the optimized algorithm’s performance was evaluated.

### Image acquisition

MR images were acquired on a clinical 3.0 T MRI scanner (Achieva, Philips). Neutral position of the supine patient was maintained by extending the knee joints with the patellae facing as anterior as possible while position was maintained by sandbags and positioning aids. The inbuilt body coil was used for streamlined image acquisition from pelvis to feet while eliminating the need for repositioning. Following a triplane localizer sequence, axial T2-weighted non-fat-saturated 2D turbospin-echo sequences were acquired in three stacks over the pelvis (both hips), knees, and ankles using the protocol detailed in Supplementary Table 4. Centered on the joint, 29 slices were acquired to fully cover each joint.

### Manual reference measurements

**Figure 2:**
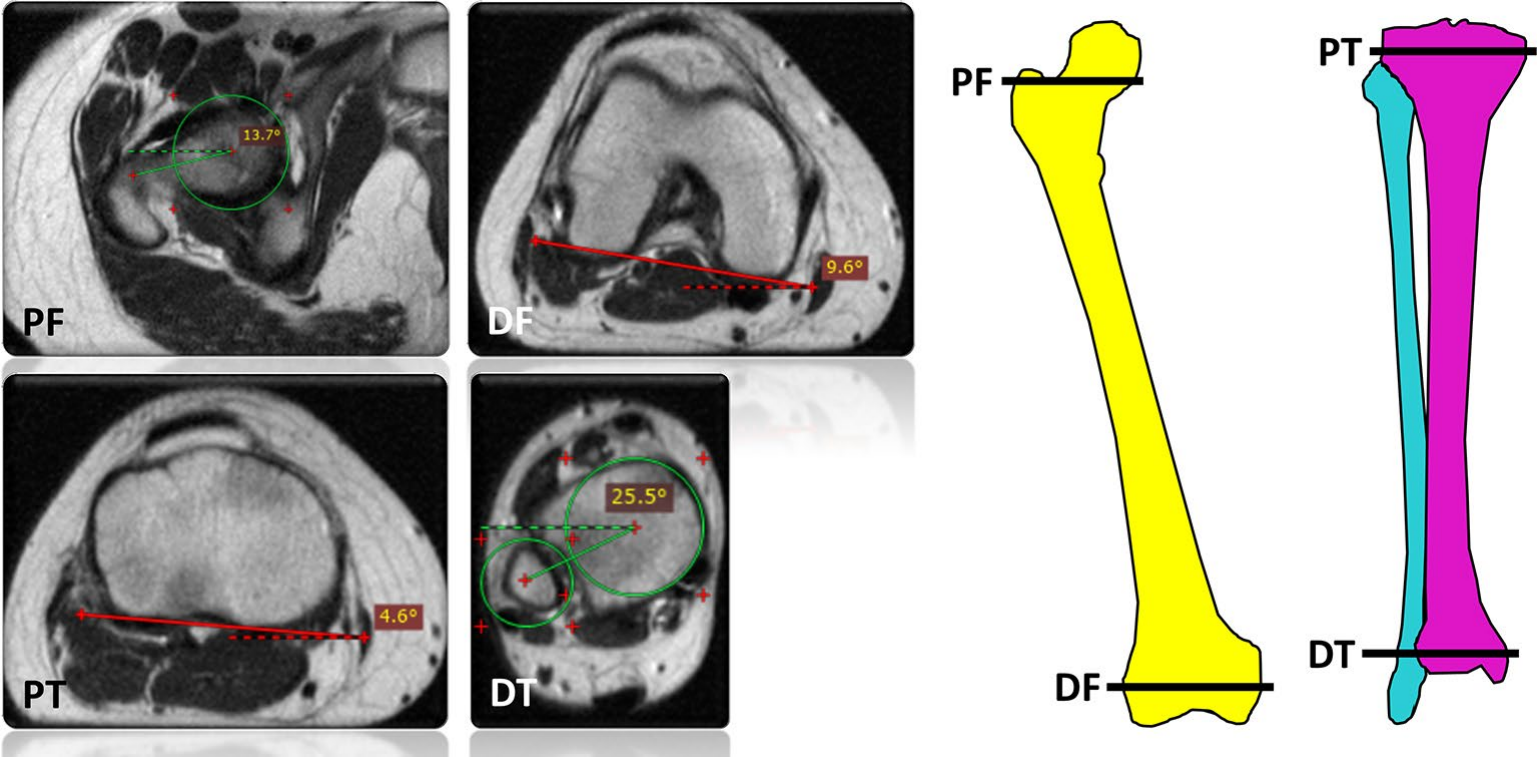
Manual reference measurements to determine femoral and tibial torsion at the levels of the hip, knee, and anke. Anatomic landmarks were used to define the reference lines at the hip in line with the method suggested by Lee (*PF* proximal femur). The reference lines at the knee were delineated as the distal femoral reference line (*DF* distal femur) and as the proximal tibial reference line (*PT* proximal tibia), while the reference line at the ankle was determined using the ellipses method (*DT* distal tibia). The circles indicate the (superimposed) femoral head and ellipses along the medial malleolus and fibular notch, while dotted lines visualize the horizontal reference lines. Schematics of femur (yellow), tibia (purple), and fibula (light blue) on the right indicate the levels of the axial images. 12-year-old female. Please refer to Supplementary Figure 3 for a visualization of the other manual reference methods to determine femoral torsion (according to the Reikeras, Tomczak, and Murphy methods) and tibial torsion (according to the bimalleolar and talus methods).

Two clinical radiologists (M.H. and S.N., with 1 and 8 years of experience in musculoskeletal imaging) performed the manual reference measurements on the anonymized test set using the RadiAnt DICOM Viewer (version 2020.1.1, Medixant) and its standard image analysis toolbox. Established methods of reference were chosen as visualized in Fig. 2 and Supplementary Figure 3. At the hip, the reference lines were defined from proximal to distal according to Lee^28^, Reikerås^30^, Tomczak^31^, and Murphy^29^, while at the knee, the posterior femoral and tibial condyles were connected^33^. At the ankle, reference lines were identified using the ellipses, bimalleolar, and talus methods^8,36,37^. The individual methods to define the reference lines are described in Table 3.

**Table 3:** Description of the methods for manual definition of femoral and distal reference lines as modified from^8,10^.

Bone level	Method	Axial image	Description of reference line
Hip/proximal femur	Lee et al.^14^	That visualizes femoral head center, femoral neck, and cephalic junction of greater trochanter (most proximal)	Connection between femoral head center and femoral neck center (single image)
	Reikerås et al.^15^	That visualizes the femoral neck at the level where the anterior and posterior cortices run parallel	Connection between femoral head center and femoral neck axis (superimposed image)
	Tomczak et al.^16^	That visualizes the greater trochanter at the level of the base of the femoral neck	Connection between femoral head center and midpoint of the greater trochanter (superimposed image)
	Murphy et al.^17^	That visualizes the femoral neck's base just cranial to the lesser trochanter	Connection between femoral head center and midpoint of the femoral neck's base
Knee/distal femur	Posterior Condyles^18^	That visualizes the largest diameters of the femoral condyles	Connection between the medial and lateral posterior facets of the femoral condyles
Knee/proximal tibia	Posterior Condyles^18^	That visualizes the tibial condyles just cranial of the fibular head	Connection between the medial and lateral posterior facets of the tibial condyles
Ankle/distal tibia	Ellipses (‡)^8,20^	That visualizes the most distal tibia	Connection of the centers of two ellipses along the medial malleolus and along the fibular notch
	Bimalleolar^8,15^	That visualizes the medial and lateral malleoli and the talar dome	Connection of the centers of the articulating cortices of the medial and lateral malleoli
	Talus^19^	That is just caudal of the tip of the medial malleolus	Line along the anterior surface of the talus

For the reference lines at the knee and ankle, descriptive terminology (instead of the first descriptor’s personal name) was used because of as-yet equivocal terminology. Please note that the ellipses method is also referred to as the Ulm method or Waidelich method (‡)^8,36^ and that the algorithmic implementation was most similar to the Lee and ellipses methods.

Projected angles between the proximal and distal reference lines were determined to calculate torsion. Internal (or external) rotation of the distal relative to the proximal bone is reflective of antetorsion (or retrotorsion)^9^. Also, radiologists’ time demand for bilateral measurements of femoral and tibial torsion based on the Lee and ellipses methods was measured on eight random MRI studies.

### Manual segmentations

The bone contours of the femur, tibia, and fibula were manually delineated on all slices as ‘ground truth’ segmentations. DN (student of the imaging sciences) labeled the bone contours using ITK-SNAP software (v3.8, Cognitica; https://www.itksnap.org) and its function of semiautomatic segmentation^39^ after being trained on five MRI studies by an experienced musculoskeletal radiologist (SN) who also reviewed all segmentation outlines for accuracy and consistency.

### Algorithm-based segmentations

**Figure 3:**
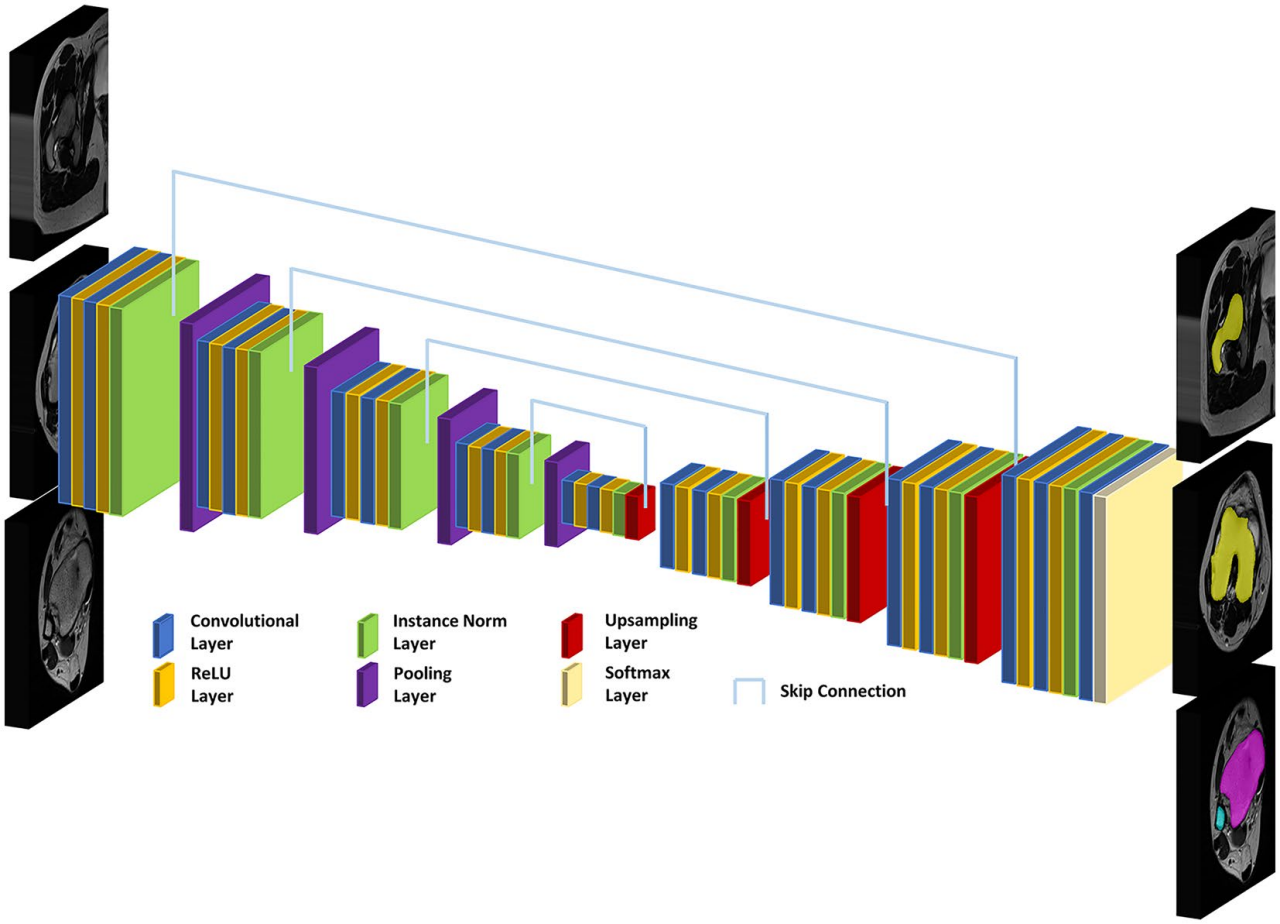
Schematic visualization of the U-net convolutional neural network used for automatic segmentations of femur (yellow), tibia (purple), and fibula (light blue). Global information were compressed to a more compact global representation. Local information was preserved by skip connections which passed the more detailed images directly to the decoder path. Up-sampling was performed by bilinear interpolation followed by 1 × 1x1 convolutions. The rectified linear unit (ReLU) was applied in all layers except the last, where a Softmax layer determined each voxels’ class probabilities. 3D convolutions aimed to reflect the high dimensionality of the MRI data. Instance normalization was chosen due to small batch size.

For automatic segmentations, we used a U-net convolutional neural network as suggested previously^40^. At a depth of 6, the network was designed with 16 filters per convolution that doubled with each pooling layer (Fig. 3). Minor modifications to the original network architecture^40^ included (i) instance normalization instead of batch normalization (due to small batch sizes), (ii) trilinear interpolation for up-sampling followed by 1 × 1 × 1 convolution instead of transposed convolutions (to reduce parameter numbers), and (iii) 3D convolutions instead of 2D convolutions (to reflect the higher dimensionality of the MRI data). Data pre-processing was implemented based on a self-adapting framework that yields excellent segmentation results^24^ and included resampling to the median voxel spacing of 0.6 × 0.6 × 6.5 mm^3^, cropping, and padding. The voxels’ signal intensity distribution was normalized to a mean of 0 and a standard deviation of 1 across the whole image.

During training, modelled predictions of segmentations were compared to the ground truth by employing a combination of the soft Dice loss on all foreground classes, i.e., femur, tibia, and fibula, and the categorical cross-entropy loss on all classes^24^. Calculated loss values were used to optimize the network parameters using stochastic gradient descent optimization with *adaptive moment estimation* (Adam)^41^ and a learning rate scheduling system that avoids local minima by decreasing the learning rate by two on training plateaus through monitoring of the moving training loss average. To avoid overfitting, data were augmented by including a random choice of motion, ghosting, spiking, and bias-field artifacts^42^. Training was performed on a *state-of-the-art* graphic-processing-unit (GeForce RTX 3090, NVIDIA) with a batch size of 1 and an initial learning rate of 0.001.

### Algorithm-based analysis of torsion

**Figure 4:**
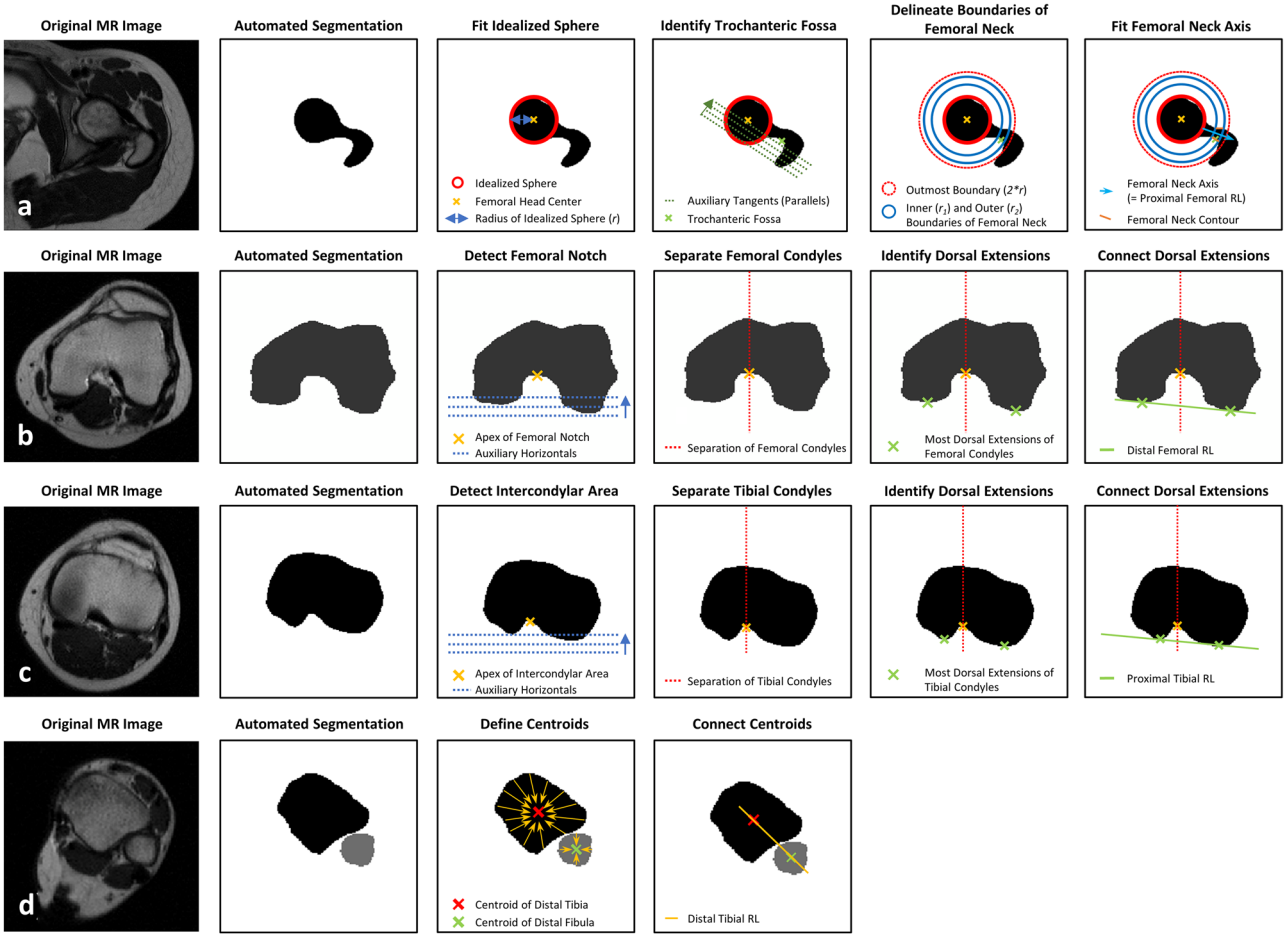
Algorithm-based identification of proximal and distal femoral and tibial reference lines to determine lower limb torsion. (**a**) At the hip, the femoral head centre was identified and used to define the femoral neck axis that served as the proximal femoral reference line. (**b**, **c**) Around the knee, the most posterior extensions of the medial and lateral femoral and tibial condyles were identified and connected as the distal femoral (**b**) and proximal tibial reference lines (**c**). (**d**) At the ankle, the tibial and fibular centroids were connected as the distal tibial reference line. *RL* reference line. MR images are not to scale.

**Figure 5:**
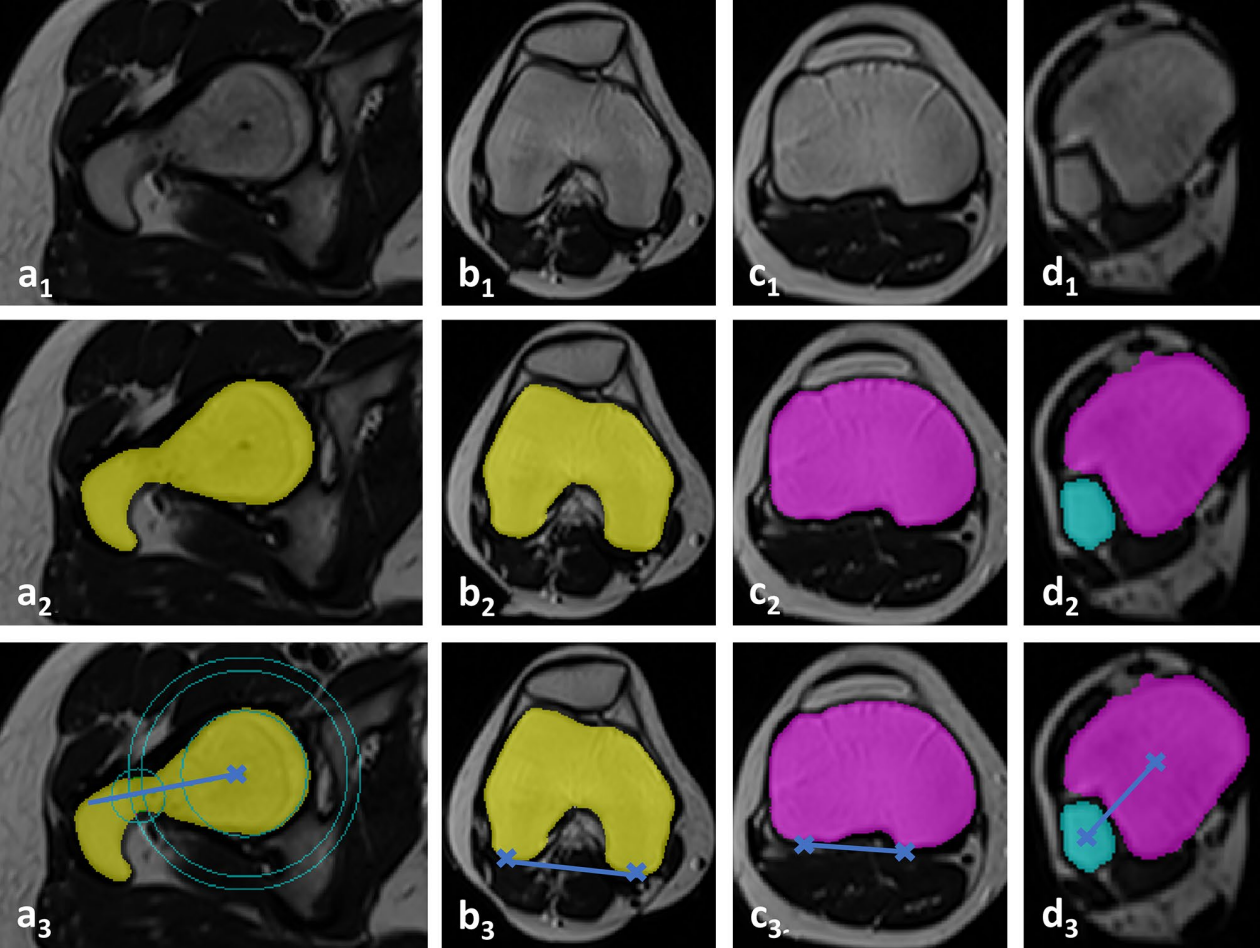
Visualization of the algorithm-based identification of the reference lines. Indicated are the original MR images (a_1_-d_1_), their segmentation outlines on selected axial images (a_2_-d_2_), and the corresponding reference lines (a_3_-d_3_) as determined by the algorithm. Proximal femur (**a**), distal femur (**b**), proximal tibia (**c**), and distal tibia (**d**). Reference lines and points are blue. For algorithmic details please refer to Fig. 4. Femur (yellow), tibia (purple), and fibula (light blue). 19-year-old female.

After segmentations, femoral and tibial torsion were determined based on the proximal and distal reference lines and used to quantify each bone’s torsion. The algorithmic procedures are schematically visualized in Fig. 4 and described in detail in the Supplementary Text 1. In a representative patient, Fig. 5 visualizes the original MR images, the automatic segmentation outlines, and the corresponding reference lines.

### Algorithm availability and time demand

The algorithm is publicly available on GitHub (https://github.com). Time demand for analysis of torsion in one lower limb (consisting of three MRI stacks) was determined on a specialized workstation with a dedicated graphic-processing-unit (Intel-Core i7-9700 K@3.60 GHz, GeForce RTX 2080 Ti, NVIDIA) and evaluated against the manual reference measurements using one-way analysis of variance (ANOVA).

### Statistical analysis

Statistical analysis was performed by J.S. and S.N. using the Python libraries *statsmodels* and *NumPy*, GraphPad Prism software (version 9.1.1), and R software (version 4.0.2). The Sørensen-Dice coefficient quantified correspondence of manual and automatic segmentation outlines of femur, tibia, and fibula. The D’Agostino-Pearson omnibus normality test was used to confirm underlying normality in torsional values. Torsional angles are reported for each reader, i.e., radiologist 1 (R1), radiologist 2 (R2), and the algorithm (Alg), and for the different methods that were used to select the proximal femoral and distal tibial reference lines. Absolute inter-reader differences were determined using absolute values. Inter-reader correlations and agreement were determined using Pearson’s correlation coefficient *r* and the intraclass-correlation-coefficient (ICC, single scorings, not adjusted). Repeated measures ANOVA followed by Tukey’s *post-hoc* tests were used for pairwise inter-reader comparisons. Multiplicity-adjusted *p*-values are reported throughout to account for multiple comparisons against the family-wise alpha error threshold of *p* ≤ 0.05.

## Supplementary Information

Supplementary Information.

## Data Availability

The main data supporting the results in this study are available within the paper and its Supplementary Information. Any additional datasets generated and analyzed in this study are available from the corresponding author on reasonable request.

## Code availability

The software routines developed, validated, and implemented are publicly available on GitHub (https://github.com). Any other data for research purposes are available from the corresponding author upon reasonable request.
