# Supplementary material for: Artificial intelligence-based automatic assessment of lower limb torsion on MRI
Source: Sci Rep. 2021 Dec 1;11:23244. doi: 10.1038/s41598-021-02708-y (PMC8636587; doi:10.1038/s41598-021-02708-y)
Supplement: Supplementary file 1 — Supplementary Information. [file 41598_2021_2708_MOESM1_ESM.pdf]

Supplementary Material

Title: Artificial Intelligence-Based Automatic Assessment of Lower Limb Torsion on MRI
Authors: Justus Schock, Daniel Truhn, Darius Nürnberger, Stefan Conrad, Marc Sebastian Huppertz, Sebastian Keil, Christiane Kuhl, Dorit Merhof, Sven Nebelung
Journal: Scientific Reports

Supplementary Text

Supplementary Text 1 - Detailed Description of the Algorithm

1) Algorithm-Based Analysis of Femoral Torsion

Following segmentation, analysis of femoral torsion required identification of the proximal and distal femoral reference lines.

1.1) Identification of the Proximal Femoral Reference Line

At the hip, the femoral head center was determined by iteratively fitting a sphere to the proximomedial femur by using the following approximation:

$$min \sum_i \left(r - \sqrt{(x_i - x_c)^2 + (y_i - y_c)^2 + (z_i + z_c)^2}\right)^2_{r, x_c, y_c, z_c}$$

where r, x_c, y_c, and z_c are the variables to be optimized, i.e. r is the radius and x_c, y_c, and z_c are the coordinates of any contour point of the idealized sphere, while x_i, y_i, and z_i are the coordinates of the segmented contours. Using least-square errors fitting, the femoral head center was identified as the center of the idealized sphere. The femoral neck axis was determined by identifying the most proximal image that visualized the femoral head center, the femoral neck, and the cephalic junction of the greater trochanter as a single segmentation area. Then, the trochanteric fossa was identified as the characteristic extra-articular depression medial of the greater trochanter that indicates the dorsal and lateral femoral neck (1): Auxiliary tangents that connected the most posterior extension of the femoral head and the most posterior extension of the greater trochanter were shifted parallelly until the trochanteric fossa was identified as a characteristic notch in the segmentation outline with a width of ≥five pixels. The distance between the trochanteric fossa and the femoral head center was determined (in the following connoted as D) and used for subsequent plausibility analyses. Two auxiliary circles with defined radii ($r_1 = 0.9 * D; r_2 = 1.1 * D$) were circled around the femoral head center to define the peripheral femoral neck. *Post hoc*, r_1 and r_2 were considered plausible if they were within the range defined by r and $2 * r$ (of the idealized sphere). Last, the peripheral femoral neck as bounded by both circles and the segmented ventral and dorsal bone outlines was subject to least-square errors fitting to define the femoral neck axis in-between the ventral and dorsal neck contours as the **proximal femoral reference line**. This procedure is schematically visualized in **Figure 4a**.

1.2) Identification of the Distal Femoral Reference Line

At the knee, the distal femoral reference line was determined as follows: First, the axial image with the largest convex area of segmented femur was selected. Second, the image

was rotated to align the most posterior extensions of the medial and lateral femoral condyles horizontally. Third, the apex of the intercondylar femoral notch was detected and used to separate the medial and lateral femoral condyles. To this end, the segmentation areas of the femur were screened from posterior to anterior using auxiliary horizontals to identify the notch apex as the most ventral non-segmented coordinate. The most posterior extensions of the medial and lateral femoral condyles were then connected by a tangent as the **distal femoral reference line**. This procedure is schematically visualized in **Figure 4b**.

2) Algorithm-Based Analysis of Tibial Torsion

As above, analysis of tibial torsion required identification of the proximal and distal tibial reference lines.

2.1) Identification of the Proximal Tibial Reference Line

At the knee, the **proximal tibial reference line** was identified using the same approach as described above for the distal femoral reference line. Yet, instead of detecting the (femoral) notch apex, the algorithm detected the apex of the posterior intercondylar area. This procedure is schematically visualized in **Figure 4c**.

2.2) Identification of the Distal Tibial Reference Line

At the ankle, the distal tibial reference line was defined on the axial image with the largest width of the segmented tibia. Following definition of the centroids of the tibia and fibula (as the arithmetic means of both segmentation outlines), both centroids were connected as the **distal tibial reference line**. This procedure is schematically visualized in **Figure 4d**.

3) Quantification of Femoral and Tibial Torsion

Torsion angles for the proximal and distal femur and tibia were determined by referencing the proximal and distal reference lines to the horizontal line. For each bone, torsion was then quantified as the angles between the proximal and distal reference lines. In line with the manual reference measurements, external (or internal) rotation of the distal reference line relative to the proximal reference line was reflected by subtraction (or addition) of the distal from the proximal angle (2).

Literature

1. Papadakis SA, Shepherd L, Babourda EC, Papadakis S. Piriform and trochanteric fossae. A drawing mismatch or a terminology error? A review. Surgical and Radiologic Anatomy. 2005;27(3):223-6.

2. Koenig JK, Pring ME, Dwek JR. MR evaluation of femoral neck version and tibial torsion. Pediatric radiology. 2012;42(1):113-5.

Supplementary Figures

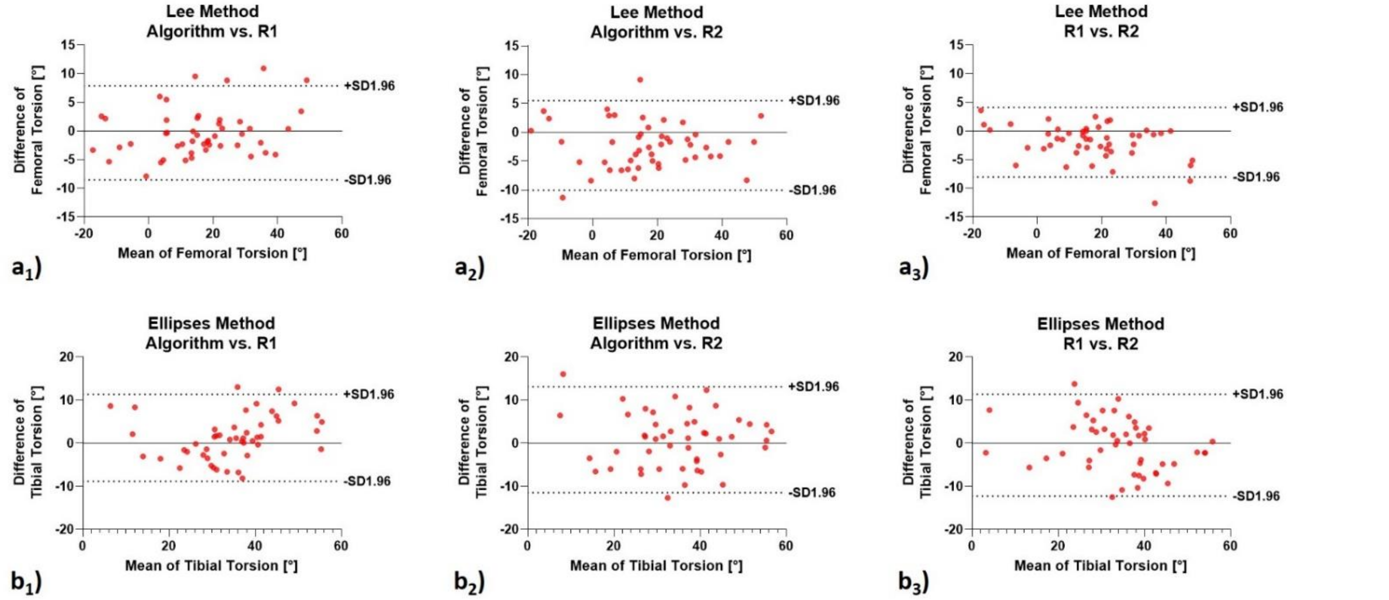

Supplementary Figure 1: Systematic inter-reader evaluation of torsional measurements.
Bland-Altman plots display the pair-wise comparisons of the readers, i.e., the algorithm, radiologist 1 (R1), and radiologist 2 (R2), for the Lee and ellipses methods that were used to define the reference lines of the proximal femur and of the distal tibia. Femoral torsion (a) and tibial torsion (b) are plotted for the algorithm vs R1 (a_1, b_1), for the algorithm vs R2 (a_2, b_2), and for R1 vs R2 (a_3, b_3). Continuous lines indicate zero, while dotted lines indicate the upper and lower limits of 95% agreement that are indicated as ±1.96 SD (standard deviation).

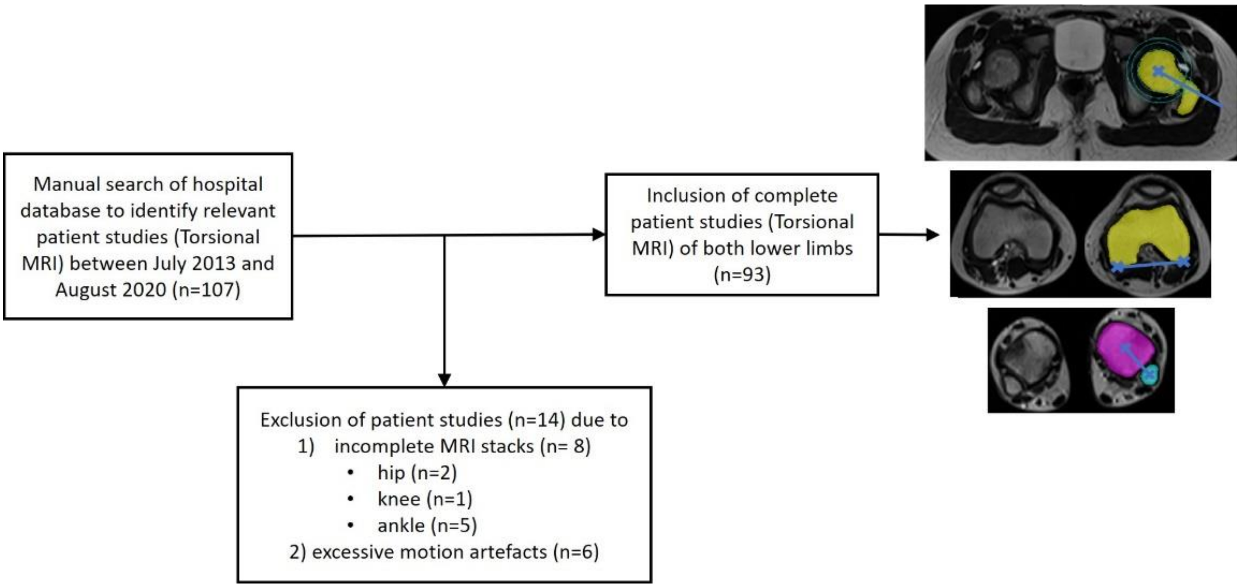

Supplementary Figure 2: Flowchart indicating numbers and details of in- and excluded patients.
Images on right indicate original MR images of the hips, knees, and ankles (left half) and outputs of segmentation and post-processing (right half).

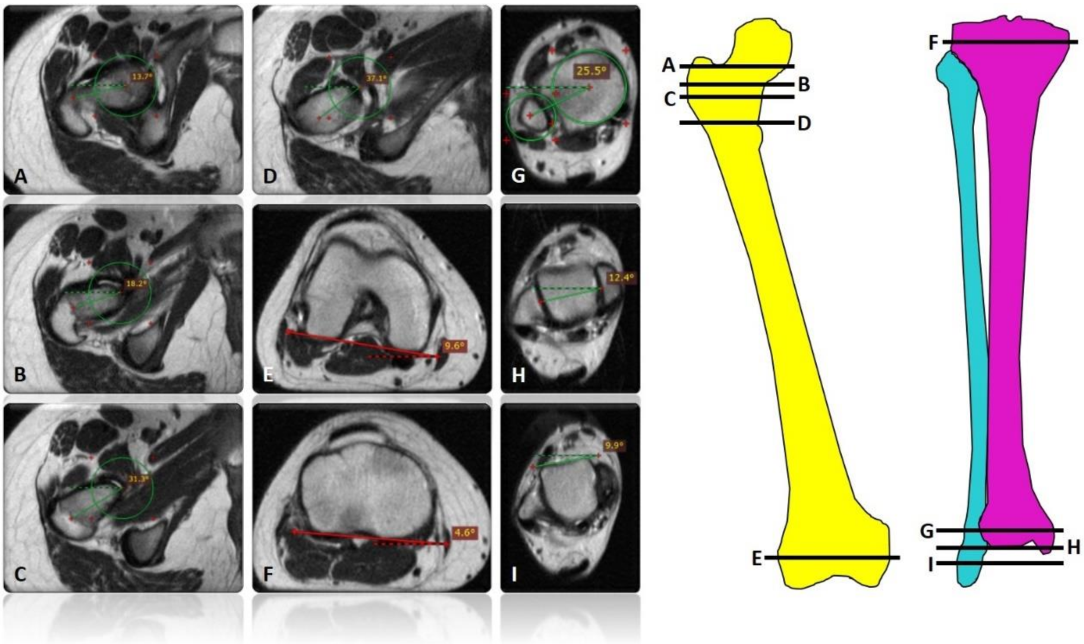

Supplementary Figure 3: Manual reference measurements to determine femoral and tibial torsion at the levels of the hip (A-D), knee (E, F), and ankle (G-I).

Anatomic landmarks were used to define the reference lines at the hip in line with the methods suggested by Lee et al. (A), Reikerås et al. (B), Tomczak et al. (C), and Murphy et al. (D). The reference lines at the knee were delineated as the distal femoral reference line (E) and proximal tibial reference line (F), while the reference lines at the ankle were determined using the ellipses (G) and bimalleolar methods (H) and along the anterior talus surface (I). Circles in A-D indicate the (superimposed) femoral head and dotted lines the horizontal reference lines. Schematics of femur (yellow), tibia (purple), and fibula (light blue) on the right indicate the levels of the axial images (A-I). 12-year-old female.

Supplementary Tables

Supplementary Table 1: Demographic Data for Patients Included in the Training, Validation, and Test Sets.

	All patients	Training Set	Validation Set	Test Set
**No. of patients**	93	60	9	24
**No. of male patients**	52	33	6	13
**Age [years]**<br>**Mean ± Standard Deviation**<br>**(Range)**	13.1 ± 5.0<br>(5-34)	13.1 ± 5.4<br>(5-34)	14.0 ± 4.6<br>(6-21)	12.8 ± 3.9<br>(5-24)

Supplementary Table 2: Mean Femoral and Tibial Torsion as a Function of Method and Reader.
Methods are differentiated by the procedure of selecting the reference line. Readers are radiologist 1 (R1), radiologist 2 (R2), and the algorithm (Alg). Means (95 % confidence intervals) [°]. Statistical analysis was performed by repeated measures ANOVA followed by pair-wise Tukey's post-hoc test. Significant differences are indicated by adjusted p-values in **bold type**.

	**Method**	**Reader**			**p-values**	**Post-Hoc Test Details**		
		**R1**	**R2**	**Alg**		**Alg vs R1**	**Alg vs R2**	**R1 vs R2**
**Femoral Torsion**	**Lee**	16.1 (11.6; 20.6)	18.0 (13.2; 22.9)	15.8 (11.0; 20.6)	<0.001	0.859	**<0.001**	**<0.001**
	**Reikeras**	21.7 (17.2; 26.2)	21.5 (16.8; 26.3)		<0.001	**<0.001**	**<0.001**	0.965
	**Tomczak**	32.7 (28.3; 37.0)	31.1 (26.4; 35.8)		<0.001	**<0.001**	**<0.001**	**0.011**
	**Murphy**	32.2 (27.3; 37.1)	31.9 (26.7; 37.2)		<0.001	**<0.001**	**<0.001**	0.845
**Tibial Torsion**	**Ellipses**	33.9 (30.8; 37.0)	34.3 (30.7; 37.9)	35.2 (31.7; 38.6)	0.311	0.212	0.622	0.877
	**Bimalleolar**	25.7 (22.3; 29.1)	26.3 (22.7; 29.9)		<0.001	**<0.001**	**<0.001**	0.611
	**Talus**	20.9 (17.1; 24.8)	21.7 (17.9; 25.6)		<0.001	**<0.001**	**<0.001**	0.566

Supplementary Table 3: Metrics of Inter-Reader Difference and Agreement for Femoral and Tibial Torsion for Different Methods.

Readers were the two radiologists (radiologist 1 [R1], radiologist 2 [R2]) and the algorithm (Alg). In a pair-wise manner, absolute differences [°] and agreement were quantified in terms of Pearson's correlation coefficient *r* and the intraclass-correlation-coefficient (ICC, single scorings [not adjusted]). Means (95 % confidence intervals). na – not applicable.

	**Method**	**Comparison**	**Absolute difference [°]**		**Pearson's r**		**ICC**	
			R2	Alg	R2	Alg	R2	Alg
**Femoral Torsion**	**Lee**	R1	2.6 (1.9; 3.3)	3.3 (2.5; 4.0)	0.984 (<0.001)	0.968 (<0.001)	0.974 (0.96, 0.99)	0.963 (0.93; 0.98)
		R2	na	3.7 (3.0; 4.5)	na	0.971 (<0.001)	na	0.966 (0.94; 0.98)
	**Reikeras**	R1	2.7 (2.1; 3.3)	6.4 (5.4; 7.5)	0.978 (<0.001)	0.964 (<0.001)	0.977 (0.96; 0.99)	0.909 (0.84; 0.95)
		R2	na	6.1 (5.0; 7.2)	na	0.967 (<0.001)	na	0.899 (0.83; 0.94)
	**Tomczak**	R1	3.2 (2.6; 3.8)	16.9 (15.7; 18.1)	0.976 (<0.001)	0.97 (<0.001)	0.967 (0.94; 0.98)	0.612 (0.4; 0.76)
		R2	na	15.3 (14.1; 16.5)	na	0.968 (<0.001)	na	0.529 (0.29; 0.71)
	**Murphy**	R1	2.6 (2.1; 3.1)	16.4 (15.1; 17.7)	0.986 (<0.001)	0.963 (<0.001)	0.984 (0.97; 0.99)	0.607 (0.39; 0.76)
		R2	na	16.2 (14.7; 17.6)	na	0.961 (<0.001)	na	0.583 (0.36; 0.74)
**Tibial Torsion**	**Ellipses**	R1	5.0 (4.0; 6.0)	4.2 (3.2; 5.1)	0.871 (<0.001)	0.904 (<0.001)	0.865 (0.77; 0.92)	0.867 (0.78; 0.92)
		R2	na	5.1 (4.1; 6.2)	na	0.867 (<0.001)	na	0.894 (0.82, 0.94)
	**Bimalleolar**	R1	3.3 (2.4; 4.2)	9.4 (8.3; 10.6)	0.933 (<0.001)	0.944 (<0.001)	0.932 (0.88; 0.96)	0.703 (0.53; 0.82)
		R2	na	9.0 (7.7; 10.3)	na	0.927 (<0.001)	na	0.679 (0.49; 0.81)
	**Talus**	R1	4.3 (3.3; 5.3)	14.2 (12.2; 16.2)	0.914 (<0.001)	0.854 (<0.001)	0.913 (0.85; 0.95)	0.451 (0.2; 0.65)
		R2	na	13.5 (11.5; 15.4)	na	0.86 (<0.001)	na	0.406 (0.14; 0.62)

Supplementary Table 4: MRI Sequence Parameters.
For stack-wise imaging of the pelvis (hips), knees, and ankles, axial T2-weighted non-fat-saturated 2D turbospin-echo sequences were acquired in three stacks.

Sequence Parameter	Detail
**Repetition Time [ms]**	3879
**Echo time [ms]**	80
**Turbo spin-echo factor**	31
**Field of view [mm]**	320 x 320
**Acquisition matrix [px]**	400 x 398
**Reconstruction matrix [px]**	528 x 528
**Pixel size [mm]**	0.8 x 0.8
**Scan percentage [%]**	100
**Flip angle [°]**	90
**Number of signal averages [n]**	2
**Slices [n]**	3 x 29 = 87
**Slice Thickness [mm]**	4.0
**Slice Gap [mm]**	0.4
**Duration [min:s]**	11:07
